# Artificial Tertiary Lymphoid Structures: Exploring Mesenchymal Stromal Cells as a Platform for Immune Niche Formation

**DOI:** 10.3390/ijms252413286

**Published:** 2024-12-11

**Authors:** Ekaterina Zubkova, Alexander Kalinin, Irina Beloglazova, Ella Kurilina, Mikhail Menshikov, Yelena Parfyonova, Zoya Tsokolaeva

**Affiliations:** 1 National Medical Research Center of Cardiology Named after Academician E.I. Chazov, Moscow 121552, Russia; 2 Faculty of Fundamental Medicine, Lomonosov Moscow State University, Moscow 119991, Russia; 3 Negovsky Research Institute of General Reanimatology, Federal Research and Clinical Center of Intensive Care Medicine and Rehabilitology, Moscow 107031, Russia

**Keywords:** artificial tertiary lymphoid structures (TLSs), mesenchymal stromal cells, immuno-organoids, TNF-alpha

## Abstract

Constructing artificial tertiary lymphoid structures (TLSs) opens new avenues for advancing cancer immunotherapy and personalized medicine by creating controllable immune niches. Mesenchymal stromal cells (MSCs) offer an ideal stromal source for such constructs, given their potent immunomodulatory abilities and accessibility. In this study, we explored the potential of adipose-derived MSCs to adopt TLS-supportive phenotypes and facilitate lymphocyte organization. Single-cell RNA sequencing revealed a distinct subpopulation of MSCs expressing key fibroblastic reticular cell (FRC)-associated markers, including IL-7, PDPN, and IL-15, though lacking follicular dendritic cell (FDC) markers. TNF-α stimulation, but not LTα2β1, further enhanced FRC marker expression (IL-7, PDPN, and ICAM1). Notably, in 3D spheroid co-culture with lymphocytes, MSCs upregulated additional FRC markers, specifically CCL21. Upon implantation into adipose tissue, MSC-lymphocyte organoids maintained structural integrity and showed extensive T-cell infiltration and partial vascularization after 15 days in vivo, although organized B-cell follicles and FDC markers were still lacking. These findings highlight MSCs’ intrinsic ability to adopt an FRC-like phenotype that supports T-cell and HEV organization, suggesting that further optimization, including genetic modification, may be needed to achieve an FDC phenotype and replicate the full architectural and functional complexity of TLSs.

## 1. Introduction

The tumor microenvironment (TME) plays a critical role in determining cancer progression and therapeutic response. Among the key immune-regulatory components within the TME are tertiary lymphoid structures (TLSs), which arise through lymphoid neogenesis in response to chronic inflammation. These ectopic lymphoid aggregates form in various non-lymphoid tissues, including tumors, and function as hubs for immune cell activation, antigen presentation, and adaptive immune responses, closely mirroring the architecture and function of secondary lymphoid organs [1].

Historically, TLSs were seen as pathogenic due to their association with chronic inflammatory diseases and cancer. However, recent studies reveal a more nuanced role, particularly in the tumor microenvironment, where TLSs can enhance anti-tumor immune responses. They recruit and retain immunoregulatory cells, such as T regulatory cells (Tregs), B-cells, innate immune cells, and stromal cells. Notably, the presence of TLSs in cancers like ovarian, melanoma, breast, colorectal, and non-small cell lung cancer correlates with improved patient outcomes and better responses to immune checkpoint inhibitors.

The formation of TLSs involves complex cellular interactions and cytokine signaling pathways. Key cytokines like TNF-α and lymphotoxin-α1β2 initiate and sustain TLS formation by driving the differentiation of lymphoid tissue organizer (LTo) cells and promoting the secretion of chemokines such as CXCL13, CCL19, and CCL21. These chemokines are essential for recruiting lymphocytes and structuring them into organized immune aggregates [2].

TLS formation also depends heavily on a stromal backbone, which supports the architecture of lymphoid tissue and sustains immune cell localization and activation [2]. In mice and humans, lymph node development is driven by prenatal interactions between lymphoid tissue inducer (LTi) cells and mesenchymal PDGFRα+ PDGFRβ+ LTβR+ LTo cells [2]. In TLS formation, infiltrating lymphocytes act as lymphoid tissue initiator (LTi) cells, particularly in perivascular areas where they interact with fibroblasts and stromal–vascular PDGFRβ+ cells [3,4].

Under inflammatory conditions, resident PDGFR+ MSCs can adopt an LTo-like phenotype, expressing LTβR, CCL19, and ICAM1, which are crucial for recruiting immune cells and assembling TLSs [5]. MSCs share functional characteristics with fibroblastic reticular cells (FRCs), marginal reticular cells (MRCs), and follicular dendritic cells (FDCs), all vital in maintaining lymphoid architecture. These cells, including MSCs, express markers like CD73, CD90, CD105, PDGFRα, and Sca-1 [6] and modulate T-cell responses, enhancing proliferation and differentiation into functional subsets critical for anti-tumor immunity.

Adipose tissue-derived mesenchymal stromal cells (AT-MSCs) are particularly noteworthy as whole adipose tissue is considered a tertiary lymphoid organ. Structures like fat-associated lymphoid clusters and milky spots easily develop within adipose tissue, providing niches for immune cell interactions and playing roles in chronic inflammation and immune responses, similar to lymph nodes but in ectopic locations [7].

Given the beneficial role of TLSs in cancer resolution, the development of artificial TLSs for immunotherapy is a promising field. Several studies have explored innovative strategies to induce TLS formation both in vitro and in vivo. Techniques include using gel-trapped lymphorganogenic chemokines to activate the LTBR-chemokine signaling axis, which promotes TLS formation in murine models, enhancing adaptive immune responses [8].

Other strategies involve implantable or injectable preparations that deliver LTBR ligands via biocompatible matrices or nanoparticles to reprogram the TME and boost local antitumor immunity (for review—[9,10]). For instance, LTBR ligand targeting has successfully induced TLS formation in pancreatic neuroendocrine tumors and glioblastoma, showcasing the therapeutic potential of TLS-based interventions [11]. Additionally, artificial TLSs have also been generated using lymph node-derived stromal cell lines, mimicking natural TLS organization with distinct B- and T-cell zones [12]. However, inducing TLS formation in vivo through the administration of high doses of gel-embedded inflammatory cytokines carries significant risks, such as severe inflammation and off-target effects. A safer and more controlled approach involves the in vitro construction of TLSs, which can then be transplanted into experimental models or patients. This method allows precise modulation of immune responses while minimizing systemic side effects.

Beyond cancer, artificial TLSs hold potential for treating chronic infections, autoimmune diseases, and serve as a platform for studying immune responses in a controlled environment. However, a critical challenge in constructing artificial TLSs lies in identifying an ideal stromal component.

While previous studies have focused on fibroblasts and dendritic cells in TLS assembly, the role of resident MSCs remains underexplored.

Adipose tissue-derived MSCs are a promising candidate due to their accessibility, ease of expansion even from small tissue samples, and well-known immunomodulatory properties. Moreover, MSCs are hypothesized to contribute to the stromal framework in natural TLS formation [5].

Our study utilized the immunomodulatory and organizational capabilities of AT-MSCs to construct TLS-like organoids. We examined the roles of TNF-α in this process, advancing our understanding of TLS formation and paving the way for clinical applications aimed at improving immune regulation in the TME and supporting cancer treatment strategies.

## 2. Results

MSC populations are known to be highly heterogeneous, even though they uniformly express surface markers CD73 (NT5E), CD90 (THY1), and CD105 (ENG).

To investigate whether MSCs contain subpopulations that resemble fibroblastic reticular cells (FRCs) or follicular dendritic cells (FDCs) typically found in TLSs, we employed single-cell RNA sequencing (scRNA-seq)—a powerful technique for dissecting cellular heterogeneity at the transcriptomic level.

### 2.1. Investigating the Potential of MSCs to Express FRC and FDC Markers

**Figure 1:**
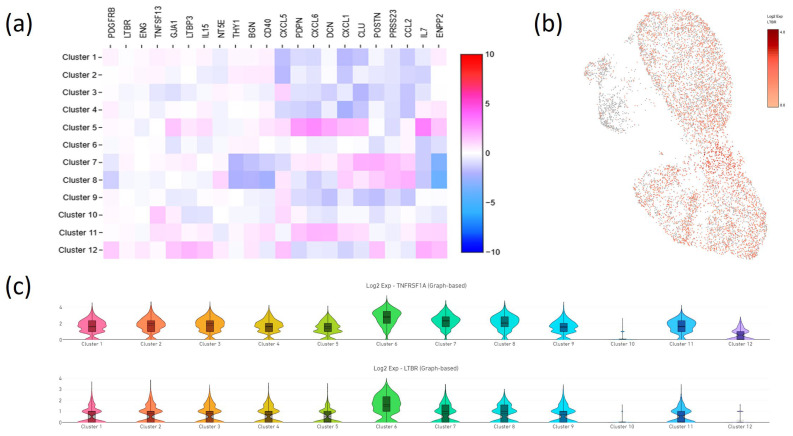
scRNA-seq analysis of human subcutaneous adipose tissue-derived MSCs. (**a**) Heat map showing the distribution of selected gene expression across clusters, with high expression levels in red and low levels in blue; (**b**) UMAP plot illustrating the distribution and expression intensity of LTBR across cells; (**c**) Violin plots showing the distribution of LTB receptor and TNF receptor 1 (TNFRSF1A) genes in each cluster. The height of the violin represents the range of gene expression values, and the width of the violin represents the density of cells showing specific expression levels in the cluster.

Our scRNA-seq analysis revealed that MSCs could be divided into 12 distinct clusters. Notably, few clusters (#5 and #12 in Figure 1) expressed key FRC-associated markers such as podoplanin (PDPN), interleukin-15 (IL15), and interleukin-7 (IL7). However, none of the MSCs expressed classical TLS markers like lymphotoxin-α (LTA), lymphotoxin-β (LTB), CD35, CCL19, CCL21, CXCL13, IL10, or CD45. These findings are visualized in a heatmap (Figure 1), along with general MSC and fibroblast markers. Interestingly, nearly all MSCs expressed mRNA for receptors crucial for TLS induction—lymphotoxin beta receptor (LTBR) and tumor necrosis factor alpha receptor 1 (TNFRSF1A)—as depicted in violin plots and UMAP visualizations (Figure 1). These data suggest that while MSCs do not naturally exhibit a full FRC or FDC phenotype, they may be primed to adopt a lymphoid tissue organizer (LTo)-like phenotype under specific cytokine stimulation.

Previous studies have demonstrated that the stimulation with TNF-α and lymphotoxin-α1β2 can induce an lymphoid tissue organizer (LTo)-like phenotype in MSCs, highlighting their potential to contribute to artificial TLS formation when exposed to specific cytokine environments [5]. To explore this further, we analyzed phenotype of MSCs cultured for 15 days in the presence of recombinant TNF-α and lymphotoxin-α2/β1 using flow cytometry. Mouse MSCs were selected for this experiment to enable direct comparison with lymph node fibroblasts isolated from the same inguinal adipose tissue depot. Simultaneous collection of human lymph nodes and adipose tissue from the same donors was not feasible, necessitating the use of mouse cells.

**Figure 2:**
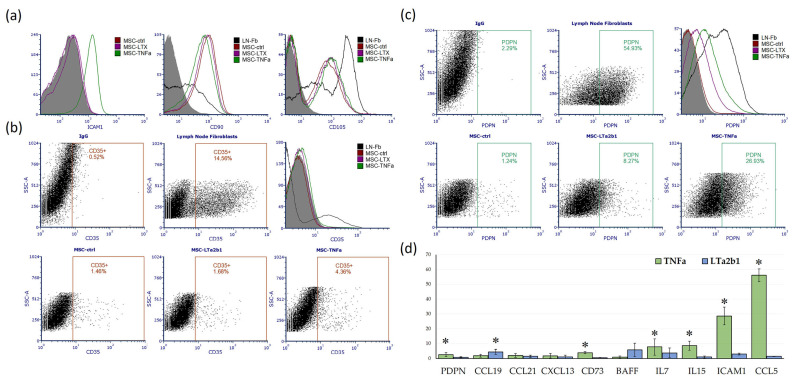
Effects of TNF-α and LTα2β1 on FRC/FDC Marker Expression. (**a**) Flow Cytometry Histograms showing surface expression of ICAM-1, CD90, and CD105 on MSCs following 15 days of incubation with 5 ng/mL TNF-α or 10 ng/mL LTα2β1 (colored histograms), on untreated lymph node fibroblasts (LN-Fb) (black histogram) and isotype control (gray histogram); (**b**) Scatter Plots and Histograms illustrating CD35 expression in untreated lymph node fibroblasts (positive control) and MSCs treated with cytokines; the percentage of CD35+ cells is indicated for each condition; (**c**) Dot Plots and Histograms of podoplanin (PDPN) expression in MSCs under different treatment conditions (colored histograms) and in untreated lymph node fibroblasts (LN-Fb) (black histogram) and isotype control (gray histogram); (**d**) qPCR Analysis of relative mRNA expression levels of selected markers (PDPN, CCL19, CCL21, CXCL13, CD73, BAFF, IL7, IL15, ICAM1, and CCL5) in MSCs following TNF-α or LTα2β1 treatment for 15 days. Statistically significant differences are indicated by an asterisk (* *p* < 0.05).

Flow cytometry confirmed MSC identity through high expression of the characteristic markers CD90 and CD105 (Figure 2a). Lymph node fibroblasts, while also expressing these markers, displayed slightly lower level of CD90 and higher of CD105 compared to MSCs, indicating subtle phenotypic differences between the two stromal cell types (Figure 2a). MSCs treated with TNF-α showed markedly elevated ICAM1 expression compared to both untreated controls and cells treated with LTα2β1 (Figure 2a).

CD35, a marker typically associated with follicular dendritic cells (FDCs), was expressed in lymph node fibroblasts (positive control) but showed only marginal expression in MSCs. TNF-α treatment led to a slight, non-significant increase in CD35 expression on MSCs, suggesting that while TNF-α may have some impact, it is insufficient to fully induce an FDC-like phenotype (Figure 2b).

Podoplanin (PDPN) expression, a marker of fibroblastic reticular cells (FRCs), was observed at high levels in lymph node fibroblasts and was significantly upregulated in MSCs treated with TNF-α, with only minimal effects from LTα2β1 (Figure 2c).

Overall findings revealed that TNF-α, but not LTα2β1, significantly enhanced the surface expression of ICAM1 and PDPN, both critical adhesion molecules that facilitate immune cell interactions [13,14].

To further support our flow cytometry findings, we performed qPCR analysis on MSCs treated with TNF-α or LTα2β1 (Figure 2d). TNF-α treatment significantly upregulated the expression of IL7, IL15, CCL5, ICAM1, PDPN, and CD73. Notably, CD73 is not only a marker of MSCs but also plays a significant role in inflammatory responses due to its enzymatic activity [15].

LTα2β1 stimulation, by contrast, led to a significant increase in CCL19 expression and resulted in a non-significant increase of up to four-fold in IL7 and BAFF expression. However, high variability in its expression levels limited statistical significance.

It is noteworthy that, the expression levels of CCL19, LTB, CR2 (encoding CD35/CD21 in mice), and CXCL13 were very close to the detection threshold, with Ct values of 34–38 out of 40 cycles. These high Ct values suggest low expression levels, potentially limited to a minor MSC subpopulation. Importantly, neither TNF-α nor LTα2β1 stimulation induced the expression of lymphotoxin-β (LTB).

Given our findings that MSCs express certain FRC markers and that this expression is enhanced by TNF-α, we focused on optimizing the conditions for organoid assembly. Spheroid formation is a crucial step in creating a three-dimensional (3D) microenvironment that closely mimics the structural and functional dynamics of natural lymphoid tissues. This approach not only facilitates enhanced cell–cell interactions but also supports the spatial organization necessary to replicate the complexity of TLSs.

**Figure 3:**
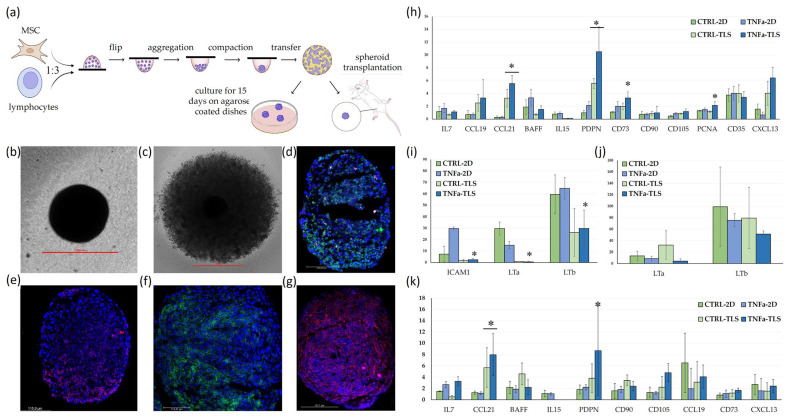
MSC-Lymphocyte Organoid Formation and Analysis of Marker Expression. (**a**) Schematic Overview of Experimental Setup. Human or mouse MSCs and lymphocytes were mixed at a 1:3 ratio and initially cultured in hanging drops for one day to promote spheroid formation. Following this compaction phase, the spheroids were either transferred to agarose-coated wells, where they were maintained for an additional 15 days or implanted into mice; (**b**) Representative phase-contrast image at 100× magnification of a typical MSC-lymphocyte spheroid on agarose, showing its general morphology and structure; (**c**) An enlarged view of the spheroid (200×); (**d**–**g**) Representative immunofluorescence images of spheroids assembled with TNF-α-pretreated MSCs, illustrating marker localization within the spheroid (**d**) CD3 Staining (green) CD20 (red) identifying T and B lymphocytes, respectively, with nuclei counterstained with DAPI (blue) (200× magnification); (**e**) CD35 staining (red); (**f**) PDPN staining (green); (**g**) PAI2 staining (red); (**h**–**k**) Quantitative PCR Analysis of Gene Expression; (**h**,**i**) relative mRNA expression levels of selected genes in both 2D co-cultures and 3D spheroids in both 2D co-cultures and 3D spheroids from human MSCs pre-treated with TNF-α (5 ng/mL) or left untreated before co-culture. Data are shown as a ratio relative to control MSCs cultured without lymphocytes and normalized to both β-actin and CD45 to ensure equal lymphocyte representation. Statistically significant differences from the control 2D group (CTRL-2D) are marked with an asterisk (* *p* < 0.05); (**j,k**) Changes in relative mRNA expression levels of various genes in 2D co-cultures and 3D spheroids from mouse MSC that were either pre-treated with recombinant mouse TNF-α (5 ng/mL) or left untreated.

Due to the differences in cell size, we selected a 1:3 MSC-to-lymphocyte ratio. Organoids formed on low-adhesion substrates like agarose or poly(HEMA) exhibited irregular shapes, with lymphocytes primarily localized at the periphery. To overcome this, we adopted a hybrid approach: MSCs and lymphocytes were first compacted in hanging drops (25–30 µL) overnight to form uniformly sized spheroids. These spheroids were then transferred to agarose-coated wells and incubated for 15 days or implanted in experimental animals. In one group MSCs were pre-incubated with TNFa for 48 h before organoid assembly. The experimental setup is illustrated in Figure 3a.

After 15 days, the spheroids were frozen, sectioned, and analyzed by immunofluorescence. We observed by CD3 staining that lymphocytes were well preserved within the spheroid structure and tended to cluster in groups (Figure 3d). CD20 expression was not detected, confirming the absence of B-cells, and CD21 expression was also absent in the spheroids.

Following TNF-α stimulation, PDPN+ and PAI-2+ MSC were also visible within the spheroids (Figure 3f,g). However, extensive staining of CD35 on the lymphocytes made it difficult to detect MSCs that might also express these markers, as the strong lymphocyte signal dominated the sections (Figure 3e).

Considering these limitations, we performed PCR analysis on the organoids and corresponding 2D co-cultures, using untreated MSCs cultured without lymphocytes as a reference. To control for lymphocyte content within the spheroids, we normalized expression levels to CD45. The results are presented in Figure 3h–k. Notably, TNF-α pre-treatment of MSCs resulted in a significant upregulation of PDPN and PAI2 expression, which correlates well with the immunofluorescence analysis demonstrating increased PDPN+ and PAI2+ cells within the spheroids. Additionally, TNF-α stimulation led to a marked increase in the expression of PCNA and CD73, indicating enhanced cell proliferation and metabolic activity.

Interestingly, CCL21 expression was elevated, while IL15 expression was reduced in 3D conditions in both mouse and human spheroids, suggesting distinct cytokine regulation in 3D cultures. In human spheroids, we also observed a significant reduction in LTA and LTB expression, whereas in mouse spheroids, LTB showed a similar trend but did not reach statistical significance due to high variability. Expression levels of canonical MSC markers, CD90 and CD105, remained consistent, indicating the preservation of the mesenchymal component within the spheroids.

### 2.2. In Vivo Implantation of MSC-Lymphocyte Organoids: Assessing Marker Expression and Integration Within Murine Tissues

To facilitate long-term study of MSC-lymphocyte organoids in vivo, we developed a specialized implantation technique to ensure both retention and clear identification of the organoids within adipose tissue. Accurately identifying transplanted organoids within host tissue can be challenging, particularly as they integrate with the surrounding microenvironment. Therefore, we selected the inguinal fat depot in mice as the implantation site, given its distinct structure, which contrasts with that of the organoids. Additionally, this site contains a large lymph node with a fully developed network of blood and lymphatic vessels, providing an ideal location for studying lymphoid-like organization and vascularization.

To create an implantation site for MSC-lymphocyte organoids, we excised the native lymph node from the inguinal fat depot and implanted the organoids embedded in autologous fibrin glue within the resulting cavity. This approach ensured that the organoids were securely positioned within the adipose tissue, where they could benefit from nearby vascular and immune cell interactions. Representative images of the organoids before implantation and of the adipose tissue containing the implanted organoids after 15 days in vivo are shown in Supplemental Figure S1.

**Figure 4:**
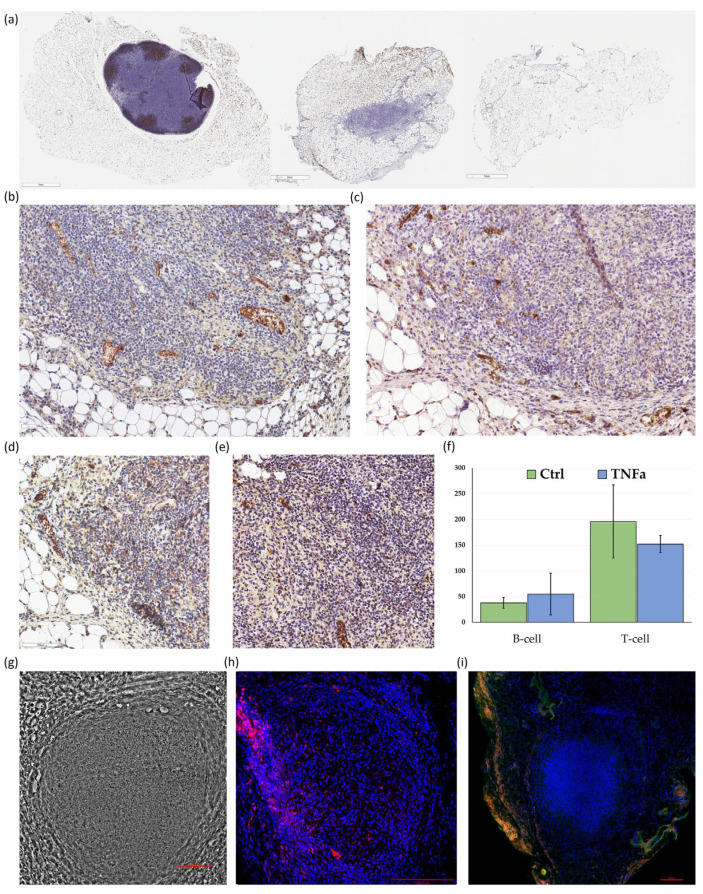
Histological and immunohistochemical characterization of implanted MSC-Lymphocyte organoids (**a**) Low-magnification immunohistochemistry images of adipose tissue cross-sections containing an intact lymph node, an implanted MSC-derived organoid, and a sham-operated control. Sections are stained for CD21 using a peroxidase-based detection system with DAB chromogen (brown) and counterstained with hematoxylin (blue) to highlight cell nuclei. Scale bars = 1 mm; (**b**) Higher magnification images of a section stained for CD21 and (**c**) CD35. Scale bars = 100 μm; (**d**) immunohistochemistry images showing CD3 and (**e**) CD20 staining, indicating the presence of T cells (CD3-positive) and B cells (CD20-positive) within the implanted organoids; (**f**) calculation of T- and B-cells in organoids. MEAN ± SD. (**g**) representative phase-contrast image showing the overall structure and cellular density of the implanted organoid at 200× magnification; (**h**) representative immunofluorescence image of an entire organoid stained with CD3 (red) to highlight T-cell distribution with nuclei stained using DAPI (blue). Scale bars = 100 μm (**i**) immunofluorescence images of frozen sections from the implanted organoid showing CD31 (green) and alpha-SMA (green) expression.

After 15 days post-implantation, the organoids were successfully recovered, displaying well-preserved structural integrity. Histological and immunohistochemical analyses showed that the organoids maintained a high cellular density, which, while lower than that observed in intact lymph nodes, was significantly greater than in the surrounding adipose tissue (Figure 4a,g). Staining for CD21 did not reveal any structures resembling B-cell lymphoid follicles within the organoids; only a few scattered CD21-positive cells were observed. Similarly, CD35 staining appeared predominantly on lymphocytes, creating challenges in detecting any follicular dendritic cell (FDC)-like structures within the organoid.

The implanted organoids showed a presence of T lymphocytes, as indicated by CD3 staining, whereas B cells (CD20-positive) appeared in notably lower numbers, typically as isolated cells scattered within the organoid structure (Figure 4d,e). No significant difference in lymphocyte counts was observed between organoids formed from TNF-α-stimulated MSCs and control organoids. This lack of a discernible difference may be due to the equal numbers of lymphocytes initially present in both organoid types, making it difficult to distinguish between newly infiltrated lymphocytes and those retained from the initial MSC-lymphocyte co-culture.

Additionally, small vessels filled with blood cells were visible within the organoid structure, indicating that the implant had undergone some degree of vascularization. However, larger vessels, particularly those positive for smooth muscle actin (SMA), were observed primarily in the surrounding tissue rather than within the organoid itself (Figure 4i).

In summary, our implantation technique successfully facilitated the localization and retention of MSC-lymphocyte organoids within the adipose tissue environment, supporting lymphocyte infiltration and partial vascularization. The MSCs exhibited characteristics similar to FRCs, including the expression of IL-7 and PDPN, and readily upregulated these factors in an inflammatory microenvironment. This FRC-like profile likely contributed to the T-cell infiltration observed within the organoids. However, the absence of organized B-cell follicles and limited expression of follicular dendritic cell markers suggest that further optimization, such as genetic engineering, may be required to fully mimic the functional architecture and immune-regulatory capacity of tertiary lymphoid structures.

## 3. Discussion

The tumor microenvironment (TME) is a highly heterogeneous and dynamic milieu where tumor cells, stromal cells, and infiltrating immune cells engage in complex interactions. This environment is commonly pro-inflammatory due to the prevalence of inflammatory cells and the secretion of pro-inflammatory cytokines and chemokines by both immune and tumor-associated cells [16]. A robust inflammatory response is a hallmark of the immune response to tumors, yet when inflammation becomes chronic and unresolved, it can drive carcinogenesis and tumor progression [17]. It is estimated that about 20% of cancers are associated with chronic inflammation linked to infections or autoimmune conditions [18]. This persistent inflammatory state in the TME contributes to tumor-supportive processes, including angiogenesis, invasion, and metastasis, which are often mediated by cytokines such as IL-6, TNF-α, and chemokines like CXCL8 [17,19].

In some cases, this chronic inflammation also promotes the spontaneous formation of tertiary lymphoid structures (TLSs) within tumors [20]. Unlike secondary lymphoid organs, TLSs are ectopic immune cell aggregates that arise in non-lymphoid tissues in response to inflammation. TLS formation is thought to have preceded the development of SLOs in evolution, likely as a mechanism to accumulate immune cells at sites of infection or injury in lower vertebrates, including amphibians and birds [21,22]. TLSs develop in stages, progressing from mixed T- and B-cell infiltrates (stage I) to structures with segregated T- and B-cell areas, lymphatic vessels, and high endothelial venules (HEVs) (stage II) and finally to mature structures with organized T- and B-cell zones, germinal centers, and follicular dendritic cells (FDCs) (stage III) [20]. Notably, TLS density in tumors is positively correlated with the infiltration of CD8+ and CD4+ T cells [23].

TLSs function as local immune niches that can stimulate adaptive immunity within the TME [24,25]. However, the precise mechanisms underlying TLS formation remain incompletely understood. There is increasing evidence suggesting that stromal elements—specifically, perivascular fibroblasts and mesenchymal stromal cells (MSCs)—play a pivotal role in organizing the architecture of TLSs [4,26]. MSCs are particularly interesting in this context as they are widely distributed, can be recruited to sites of inflammation, and possess immunomodulatory properties [27]. MSCs are also known to contribute to the maintenance of immune niches in other tissues, such as the bone marrow, where they support hematopoiesis and immune cell retention [28].

As TLSs mature, stromal cells undergo phenotypic shifts, differentiating toward fibroblastic reticular cells (FRCs) and, in more advanced TLSs, toward FDCs [25]. FRCs are known to secrete key cytokines and chemokines such as IL-7, CCL19, and CCL21, which facilitate T-cell retention and support immune interactions. FDCs, on the other hand, play a specialized role in organizing B-cell follicles and promoting humoral responses through the production of CXCL13 [29,30].

Based on this understanding, our study sought to investigate the intrinsic heterogeneity within MSC populations to assess their potential to differentiate into FRC or FDC. Using single-cell RNA sequencing, we identified a subpopulation of MSCs that expressed several FRC-associated markers, including IL-7, IL-15, and PDPN, suggesting an inherent potential for lymphoid tissue support. However, we did not detect expression of hallmark FDC markers, such as CD35, CXCL13, CCL19, or CCL21, indicating an absence of an FDC-like phenotype within untreated MSCs. While the FRC-like subpopulation was relatively small, its functional relevance was confirmed by the measurable levels of IL-7 and IL-15 in MSC-conditioned media (Supplementary Figure S2).

These findings highlight MSCs as a potential stromal source for TLS formation. Notably, MSCs expressed receptors for TNF-α (TNFRSF1A) and lymphotoxin (LTBR), positioning them as responsive to cytokine-driven differentiation signals. In line with previous studies (e.g., [5]), we observed that prolonged TNF-α stimulation, rather than LTα2β1, effectively upregulated FRC-associated genes such as IL-7, PDPN, and ICAM1 in MSCs. This response highlights the critical role of the inflammatory milieu in priming MSCs for phenotypic changes that support immune niche formation.

The flow cytometry results provided further evidence of MSCs’ adaptability under TNF-α stimulation, showing increased expression of adhesion molecules like ICAM1 and PDPN that are pivotal for cellular interactions in the immune landscape [13,14]. However, our data showed only marginal CD35 expression, indicating that TNF-α alone is insufficient to induce an FDC-like phenotype in MSCs. This result partially aligns with findings by Krautler et al., as they did not detect expression of key FDC markers in mouse adipose tissue-derived MSCs [4]. However, they reported successful FDC differentiation after transplanting MSCs into collagen sponges in immunized mice, achieving an FDC-like phenotype in 83% of transplantants over a four-week period.

The discrepancy between our findings and those of Krautler et al. may be due to differences in inflammatory conditions [4]. Krautler et al. observed successful FDC differentiation in vivo within a prolonged and robust inflammatory environment [4], likely providing additional host-derived cytokines essential for full FDC development. In contrast, our shorter TNF-α exposure led only to a modest trend toward increased CD35 expression. These findings suggest that sustained or combined cytokine stimulation may be necessary to fully induce an FDC-like phenotype in MSCs, a critical step for optimizing the functionality of artificial tertiary lymphoid structures.

Further we investigated whether different experimental conditions—cytokine stimulation, 2D co-culture with lymphocytes, and 3D spheroid co-culture—could induce MSCs to adopt features resembling lymphoid tissue organizer (LTo) or FRC/FDC phenotypes. Each approach led to distinct changes in gene expression profiles.

Cytokine stimulation alone (15-day incubation with TNF-α and LTα2β1) led to significant upregulation of markers associated with stromal and immune support, including PDPN, ICAM1, IL7, CD73, and IL15, suggesting an activation towards a lymphoid-supportive phenotype. However, in 2D MSC-lymphocyte co-cultures, where cytokine signals are naturally provided by lymphocytes themselves (e.g., LTα3, LTα2β1, and various interleukins) the effects on MSC gene expression were noticeably attenuated compared to cytokine stimulation alone. For example, ICAM1 expression, which increased approximately 30-fold under direct cytokine treatment, was only upregulated by 2.7-fold in 2D co-culture, indicating that initial TNF-α stimulation might trigger expression changes that are not sustained without continuous cytokine support.

This result was unexpected because we specifically chose not to use soluble LTα1β2 for MSC pre-treatment, relying solely on TNF-α before initiating co-culture. This decision was based on the fact that LTα1β2 naturally exists only in a membrane-bound form, expressed by activated B and T lymphocytes. Given that nearly all MSC express LTβR, the receptor for LTα1β2, we anticipated that MSCs would naturally receive lymphotoxin signaling from lymphocytes in co-culture. LTα1β2 plays a crucial role in secondary lymphoid organ development and the maintenance of FRCs and FDCs [31,32]. However, in our experimental settings, TNFα alone had a more pronounced impact on the MSC expression profile than either LTα1β2 or the natural lymphotoxin signaling provided by lymphocytes in co-culture.

The 3D co-culture condition yielded surprising results, with notable upregulation of CCL21 and PDPN. CCL21, a chemokine crucial for organizing the T-cell zone within lymphoid tissues, is secreted by FRCs to attract naive T cells and dendritic cells and facilitate immune cell interactions [33]. Additionally, PDPN, a hallmark of FRCs, supports the structural network within lymphoid tissues and modulates cell migration and adhesion dynamics [34].

These findings suggest that 3D architecture and cellular interactions in spheroids may create a more conducive environment for lymphoid-associated gene expression. The ability of 3D structures to modulate gene expression and cellular behavior is well documented in the literature [35,36]. Interestingly, in human organoids, the reduction of lymphotoxin-α (LTA) and lymphotoxin-β (LTB)—originating from lymphocytes, as MSCs have negligible production of these cytokines—were significantly reduced, hinting at a potential regulatory effect within the 3D structure that modulates lymphocyte cytokine secretion.

After testing the in vitro 2D–2D co-culture conditions, we transitioned to an in vivo model to assess the MSC-lymphocyte organoids within a host environment. To enhance transplant localization and reduce the likelihood of rejection, we designed a specific implantation method: MSC-lymphocyte organoids were embedded in mouse fibrin glue and implanted into the adipose tissue depot of mice, precisely at the site of an excised lymph node. This strategic site selection, with established lymphatic and blood vessels, offered a supportive microenvironment for vascular and lymphatic integration.

Fifteen days post-implantation, the organoids were easily identifiable within the adipose tissue, displaying notable signs of host tissue integration. Immunohistochemical analysis confirmed lymphocyte presence, primarily T cells, within the organoids. However, it remains challenging to determine whether these T cells actively infiltrated post-implantation or were retained from the initial MSC-lymphocyte preparation. Small blood vessels were present within the organoid structure, indicating early stages of vascularization, though larger smooth muscle actin (SMA)-positive vessels were primarily observed in the surrounding tissue rather than within the organoid itself, suggesting partial vascular integration.

Despite successful integration and immune cell retention, organized B-cell follicles and FDC markers were absent within the organoids, indicating that these structures resembled early-stage TLS. Although they exhibited a T cell-rich environment, they lacked mature lymphoid characteristics such as B-cell germinal centers and high endothelial venules (HEVs). These findings suggest that while MSC-lymphocyte organoids can initiate TLS-like features, further optimization may be necessary to promote the maturation of more complex lymphoid structures within the host. Importantly, developing such a model without inducing systemic inflammation remains a priority, as systemic immunization protocols or high-dose inflammatory cytokine injections, like those used in Krautler et al. [4] and similar studies, are not always feasible for translational applications. Instead, future approaches may focus on achieving local and controlled immune activation within the organoid itself, potentially through cytokine modulation or genetic engineering of MSCs to mimic key TLS-supportive signals, thus maintaining localized immune niches without systemic effects on the host.

Our results collectively suggest that MSCs can adopt an FRC-like phenotype that supports T-cell organization, positioning them as promising candidates for engineering artificial TLSs tailored to T-cell-dominant immune niches. Modeling these immune niches is vital for studying localized immune responses in complex environments, such as the tumor microenvironment or chronic inflammatory sites. Artificial TLSs enable precise examination of immune-stromal interactions, cytokine signaling, and immune cell recruitment dynamics, providing valuable insights for immunotherapy and immune-mediated disease treatments. However, the absence of FDC markers and organized B-cell structures in our models highlights a significant limitation in fully replicating the architectural and functional characteristics of natural lymphoid tissue.

Future approaches that incorporate targeted genetic modifications may enable MSCs to express additional B-cell-supportive factors, such as CXCL13, thereby enhancing their capacity to support both FRC- and FDC-like functions within artificial TLSs. This strategy has shown success in other studies, where engineered MSCs provided immune niches conducive to AML engraftment [37] or supported dendritic cell development in humanized mouse models [38]. Further programming of MSC transcriptional profiles, such as through modulation of key signaling pathways [39], could extend MSC functionality to create immune niches with full lymphoid architecture, opening new avenues for both regenerative medicine and cancer immunotherapy.

## 4. Conclusions

Our study demonstrates the potential of adipose-derived mesenchymal stromal cells as a stromal platform for engineering artificial tertiary lymphoid structures, focusing on their ability to adopt FRC-like phenotypes. Using single-cell RNA sequencing, we identified a subpopulation of MSCs expressing key FRC markers, including IL-7, IL-15, and PDPN, but lacking FDC markers such as CD35 and CXCL13. Under TNF-α stimulation, MSCs showed significant upregulation of some FRC-associated markers—PDPN and ICAM1.

In vivo implantation revealed that MSC-lymphocyte organoids implanted into the adipose tissue depot of mice supported T-cell and partial vascularization but failed to organize B-cell follicles or express FDC markers, resembling an early-stage TLS. Importantly, no inflammation was induced at the implantation site, nor were the animals subjected to treatments or disease conditions to stimulate an inflammatory microenvironment. This approach was intentionally designed to align with future clinical applications, where minimizing systemic inflammation would be essential.

These findings emphasize the intrinsic ability of MSCs to support T-cell-immune niches. However, the lack of FDC markers and B-cell follicle organization in our models underscores the need for further optimization.

## 5. Materials and Methods

### 5.1. Cell Isolation and Culturing

Small pieces of subcutaneous adipose tissue (0.5–1 mL) were obtained from volunteers (21–55 years old) after informed consent was provided. The tissue was minced with scissors, and the supernatant was used for lymphocyte isolation. The remaining tissue was digested with collagenase I (20 U/mL, Worthington Biochemical Corp., Lakewood, NJ, USA) and dispase II (5 U/mL, Sigma-Aldrich-Merck, Darmstadt, Germany) for 30–40 min at 37 °C. Following digestion, the mixture was centrifuged at 200× *g* for 10 min, and the resulting cell pellet was resuspended in DMEM containing 10% FBS (Gibco-Thermo Fisher Scientific Inc., Waltham, MA, USA). The suspension was filtered through a 40 µm mesh (Falcon Corning, Glendale, AZ, USA) and plated. Non-adherent cells were removed after 24 h. MSCs were cultured with medium changes every 2–3 days and sub-cultured at 70% confluence using 0.05% trypsin/0.02% EDTA (Gibco-Thermo Fisher Scientific Inc., Waltham, MA, USA). The MSCs were routinely characterized by flow cytometry, confirming the expression of CD73, CD90, and CD105 and the absence of hematopoietic (CD45, CD34) and endothelial (CD31) markers. Additionally, we assessed their differentiation potential using standard osteogenic and adipogenic assays: osteogenic differentiation was validated by Alizarin Red (Sigma-Aldrich-Merck, Darmstadt, Germany) staining, and adipogenic differentiation was confirmed using Bodipy (D3922, Thermo Fisher Scientific Inc., Waltham, MA, USA) or Oil Red O staining (Sigma-Aldrich-Merck, Darmstadt, Germany). Representative images have been included in Supplementary Materials Figure S3. Cells were used up to the 4th passage.

Lymphocytes were expanded in an X-Vivo 15 medium (Lonza, Basel, Switzerland) supplemented with 10 µg/mL phytohemagglutinin (PanEko, Moscow, Russia) and 5 ng/mL IL-2 (SciStore, Moscow, Russia) for 10–14 days.

For mouse MSC isolation, adipose tissue from C57BL/6 mice was processed similarly. Lymph node fibroblasts were isolated from inguinal fat depot lymph nodes through mincing, enzymatic digestion, and filtering. Non-adherent cells were removed after 24 h. Mouse lymph nodes were harvested, minced, and filtered through a 40 µm mesh for lymphocyte isolation. These lymphocytes were cultured in an X-Vivo 15 medium (Lonza, Basel, Switzerland) with IL-2 and used in co-culture experiments.

### 5.2. Organoid Formation and In Vivo Implantation

Organoids were formed by co-culturing adipose-derived MSCs and lymphocytes in a 1:3 ratio (70,000 MSCs per 30 µL drop) using a hanging drop system. After 1-day sufficient compaction occurred, resulting in spheroid formation. These spheroids were transferred to agarose-coated wells and cultured for 14 days, with media changes every 2 days.

Spheroid size and structure were routinely monitored using phase-contrast microscopy. Spheroids that were excessively small, irregularly shaped, or loosely compacted were excluded from downstream analyses to maintain consistency across experimental replicates. Organoids were subsequently analyzed by immunofluorescence and PCR. For certain experiments, MSCs were pre-incubated for 48 h with human or mouse TNF-α (5 ng/mL) (R&D Systems, Minneapolis, MN, USA).

For in vivo implantation, organoids were embedded in autologous fibrin glue. Blood was collected from mice into EDTA-coated tubes to prevent coagulation, and plasma was separated by centrifugation at 800× *g* for 15 min at 4 °C. Plasma was subjected to cryoprecipitation by freezing at −80 °C and thawing at 4 °C, a process repeated to enrich fibrinogen. Before implantation, fibrin glue was prepared by mixing equal volumes of fibrinogen concentrate with a mixture of thrombin (Calbiochem-Merck, Darmstadt, Germany), calcium chloride, and aprotinin to prevent premature fibrinolysis. This mixture was immediately used to embed the organoids, ensuring rapid polymerization upon application.

Mice were anesthetized with isoflurane, and the lymph node was surgically removed from the inguinal fat depot of the left leg. The cavity was filled with either polymerized fibrin gel alone or organoids embedded in the gel. Additional freshly prepared fibrin glue was applied on top, and the site was left for 10 min to allow full polymerization before suturing the incision. Mice were monitored daily for signs of distress. After 15 days, mice were sacrificed, and the fat tissue containing the organoids was harvested for both paraffin embedding and freezing. Two separate experiments were conducted, each with 4 animals per group.

### 5.3. Immunostaining

In vitro cultured organoids, after 15 days, were cryopreserved in Tissue-Tek O.C.T. compound (Sakura Finetek, Torrance, CA, USA), frozen in liquid nitrogen vapor, and sectioned at 7 µm thickness. For immunofluorescence, sections were fixed in 4% formaldehyde for 10 min, blocked with 5% BSA for 30 min at room temperature, and incubated overnight at 4 °C with primary antibodies against CD3 (NB600-1441SS, Novus Biologicals, Centennial, CO, USA), CD35 (A3661, Abclonal, Woburn, MA, USA), CD20 (PA5-16701, Thermo Fisher Scientific Inc., Waltham, MA, USA), CD21 (MA5-32227, Thermo Fisher Scientific Inc., Waltham, MA, USA), PDPN (120801, Biolegend, San Diego, CA, USA), CD31 (ab28364, Abcam, Cambridge, UK), and aSMA (F3777, Sigma-Aldrich-Merck, Darmstadt, Germany). After washing, sections were incubated with fluorophore-conjugated secondary antibodies (Thermo Fisher Scientific Inc., Waltham, MA, USA,) for 1 h at room temperature, mounted with VectaShield (Vector Laboratories, Inc., Newark, CA, USA), and imaged using a Stellaris 5 confocal microscope (Leica, Wetzlar, Germany).

Organoids harvested after 15 days in mouse fat tissue were either cryopreserved in Tissue-Tek O.C.T. compound (Sakura Finetek, Torrance, CA, USA) and processed for cryosectioning, as described above, or fixed in 4% paraformaldehyde, embedded in paraffin, and sectioned at 5 µm thickness. Paraffin-embedded sections were deparaffinized, subjected to heat-induced antigen retrieval in citrate buffer (pH 6.0), and blocked with 5% BSA. Sections were incubated overnight at 4 °C with primary antibodies against CD21 (MA5-32227, Thermo Fisher Scientific Inc., Waltham, MA, USA), CD3 (NB600-1441SS, Novus Biologicals, Centennial, CO, USA), CD20 (PA5-16701, Thermo Fisher Scientific Inc., Waltham, MA, USA), and CD35 (A3661, Abclonal, Woburn, MA, USA). Visualization was performed using HRP-conjugated secondary antibodies (Vector Laboratories, Inc., Newark, CA, USA) and DAB substrate (Vector Laboratories, Inc., Newark, CA, USA). Slides were counterstained with hematoxylin, mounted, and imaged with an Aperio slide scanner (Leica, Wetzlar, Germany).

### 5.4. Flow Cytometry

Mouse subcutaneous adipose tissue-derived MSCs and lymph node-derived fibroblasts were harvested and fixed with 4% paraformaldehyde for 10 min at room temperature. Following fixation, cells were washed twice with PBS. For surface marker staining, CD90, CD105, and podoplanin (PDPN, 127417, Biolegend, San Diego, CA, USA), cells were incubated with unconjugated primary antibodies diluted in PBS with 1% BSA for 30 min at 4 °C. After washing, cells were incubated with fluorophore-conjugated secondary antibodies for 30 min at 4 °C.

For CD35 staining, cells were fixed with 4% PFA and incubated with an anti-CD35 primary antibody (DF9412, Affinity Biosciences, Cincinnati, OH, USA) for 30 min at 4 °C. After washing, cells were incubated with fluorophore-conjugated (Alexa 488 or Alexa 647) secondary antibodies (Thermo Fisher Scientific Inc., Waltham, MA, USA) for another 30 min at 4 °C. The stained cells were resuspended in PBS and analyzed using a FACSCanto II flow cytometer (BD Biosciences, Franklin Lakes, NJ, USA). A minimum of 10,000 events per sample were collected. Data analysis was performed using FCS Express 7 software version 7.16.0047 (De Novo Software, Pasadena, CA, USA).

### 5.5. Single-Cell RNA Sequencing

Human subcutaneous adipose tissue-derived MSCs were isolated and cultured as described previously. At passage 2, cells were harvested for single-cell RNA sequencing (scRNA-seq). Cells were washed with PBS and dissociated using trypsin/EDTA solution (Thermo Fisher Scientific, Waltham, MA, USA) to obtain a single-cell suspension. The suspension was filtered through a 40 µm cell strainer (BD Biosciences, Franklin Lakes, NJ, USA), and cell viability was confirmed to be above 95% using Trypan Blue staining.

Single-cell capture and cDNA synthesis were performed using the 10× Genomics Chromium Single Cell 3′ v3.1 kit, following the manufacturer’s protocol. Approximately 10,000 cells were loaded per channel to achieve the capture of 7000–8000 cells. Libraries were constructed, amplified, and prepared for sequencing. The concentration of cDNA libraries was measured using the dsDNA High Sensitivity kit on a Qubit 4.0 fluorometer (Thermo Fisher Scientific Inc., Waltham, MA, USA). Library concentrations were 15.6 ng/µL with an average fragment size of 448–464 bp (ranging from 300 bp to 1000 bp). Control SI-TT-B4 (i7-GTAGACGAAA; i5a-CTAGTGTGGT; i5b-ACCACACTAG) and experimental SI-TT-C4 (i7-TTCTCGATGA; i5a-TGTCGGGCAC; i5b-GTGCCCGACA) indexes were used. Library quality was assessed using the High Sensitivity D1000 ScreenTape on the 4150 TapeStation (Agilent Technologies, Inc., Santa Clara, CA, USA).

The prepared cDNA libraries were denatured and sequenced on a NextSeq 2000 (Illumina, San Diego, CA, USA) platform to achieve a depth of 50,000 reads per cell. Sequencing data were processed using Cell Ranger software 7.0.1 (10× Genomics, Pleasanton, CA, USA) on standard settings. The processed data were visualized and analyzed using the Loupe Browser 6.4.0 (10× Genomics, Pleasanton, CA, USA), enabling detailed exploration of the single-cell transcriptomic profiles.

### 5.6. PCR Analysis

RNA was extracted using the RNeasy Mini Kit (Qiagen, Venlo, The Netherlands) following the manufacturer’s protocol. The concentration and purity of the RNA were measured using a NanoDrop spectrophotometer (Thermo Fisher Scientific Inc., Waltham, MA, USA). cDNA synthesis was performed with the High-Capacity cDNA Reverse Transcription Kit (Thermo Fisher Scientific Inc.). Quantitative PCR was conducted on a StepOnePlus Real-Time PCR System (Applied Biosystems) using an SYBR Green PCR Master Mix (EvroGen, Moscow, Russia). The qPCR cycling conditions included an initial denaturation step followed by 40 cycles of 95 °C for 15 s and 60 °C for 60 s. Beta-actin was used as the housekeeping gene, with specific primer sequences for human and mouse genes detailed in the Supplementary Materials Table S1.

Gene expression changes were analyzed using the ΔΔCt method to calculate relative fold changes.

### 5.7. Statistical Analysis

All experiments were performed in triplicate. Data were analyzed using Microsoft Excel 2016 (Microsoft Redmond, WA, USA) and GraphPad Prism 7.0 software (GraphPad Software, Inc., La Jolla, CA, USA). Results are expressed as mean ± standard deviation (SD). Statistical significance was assessed using Student’s *t*-test for comparisons between two groups with a *p*-value < 0.05 considered statistically significant.

## Data Availability

The raw data supporting the conclusions of this article will be made available by the authors on request.

## Supplementary Materials

The following supporting information can be downloaded at: https://www.mdpi.com/article/10.3390/ijms252413286/s1.
