# Supplementary material for: Artificial Tertiary Lymphoid Structures: Exploring Mesenchymal Stromal Cells as a Platform for Immune Niche Formation
Source: Int J Mol Sci. 2024 Dec 11;25(24):13286. doi: 10.3390/ijms252413286 (PMC11676966; doi:10.3390/ijms252413286)
Supplement: Supplementary file 1 [file ijms-25-13286-s001.zip › ijms-3338028-supplementary.pdf]

Supplementary

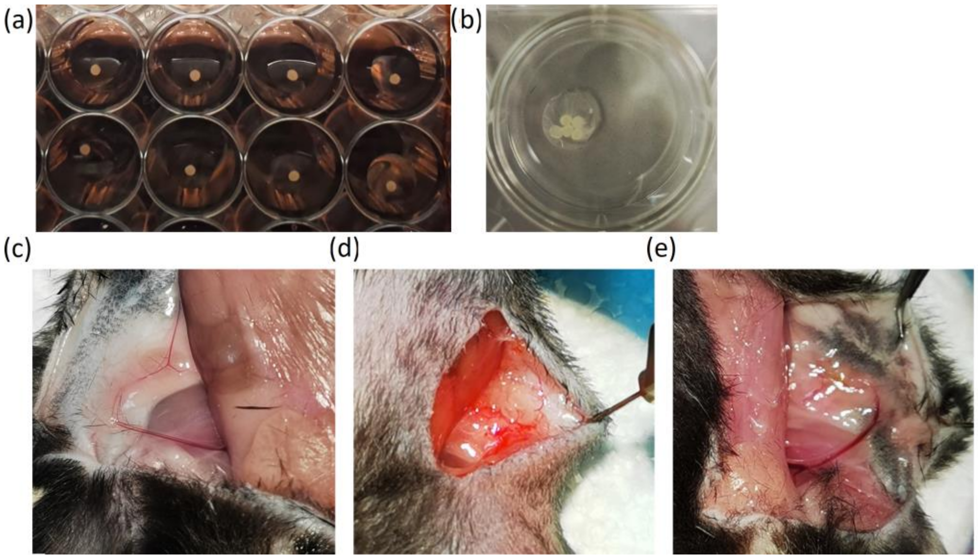

Figure S1. Preparation and transplantation of MSC-Lymphocyte organoids in adipose tissue. (a) MSC-lymphocyte organoids were assembled and cultured on low-adhesion agarose-coated plates to maintain spheroid structure and promote cell compaction before transplantation; (b) organoids embedded in autologous fibrin glue, ready for implantation; (c) intact fat depot with lymph node; (d) in vivo transplantation into the adipose tissue of the mouse inguinal depot. The organoid embedded in fibrin glue was placed into the cavity left by the excised lymph node; (e) general appearance of the adipose tissue 15 days post-transplantation.

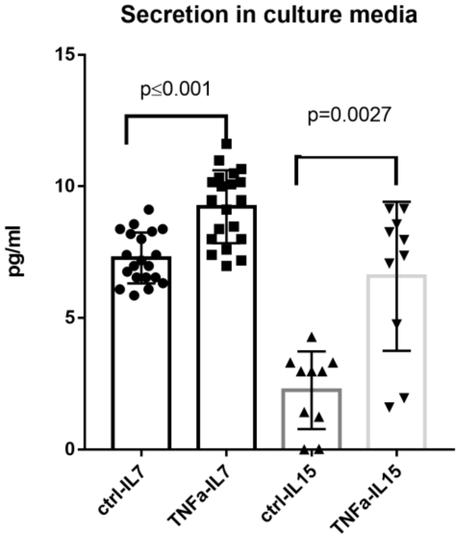

Figure S2. IL-7 and IL-15 Levels in MSC Conditioned Media Measured by Multiplex Immunoassay. IL-7 and IL-15 concentrations in conditioned media from human MSC cultures were quantified using a multiplex immunoassay (Luminex-Thermo Fisher Scientific), following the manufacturer's protocol. Briefly, supernatants were collected after 48 hours of MSC culture in serum-free medium, centrifuged to remove debris, and analyzed using Luminex xMAP technology. The assay utilized fluorescent bead-based detection, with cytokine concentrations calculated based on standard curves for each analyte. Data represent mean ± SD of three independent experiments. Statistical analysis was performed using the Mann-Whitney u-test, with $p < 0.05$ indicating significance.

Table S1. Real-time PCR primers list

	HUMAN	MOUSE
BAFF	CGTTCAGGGTCCAGAAGAAA	ACACTGCCCAACAATTCCTG
	GTCCCATGGCGTAGGTCTTA	TCGTCTCCGTTGCGTGAAATC
IL7	TGAAGGTAAAGATGGCAAACAA	TTCCTCCACTGATCCTTGTTCT
	CAATTTCTTTCATGCTGTCCAA	AGCAGCTTCCTTTGTATCATCAC
LTA	CTGCTCACCTCATTGGAGAC	CCACCTCTTGAGGGTGCTTG
	CCTGGGAGTAGACGAAGTAGAT	CATGTCGGAGAAAGGCACGAT
LTB	GGTTTCAGAAGCTGCCAGAGGA	ACGCTTCTTCTTGGCTCGC
	CGTCAGAAACGCCTGTTCCTTC	ACCTCATAGGCGCTTGGAT
CD35	GGACTGGTGCTAAGGACAGG	GGTTCGCTCTGGGTTTTCTTCAC
	ATGATGCATGTGGCAGACGA	CTGACCACTTGGAGGTTTCTAAGC
CCL19	CCAACTCTGAGTGGCACCAA	CTGCCTCAGATTATCTGCCAT
	TGAACACTACAGCAGGCACC	GTCTTCCGCATCATTAGCAC
CCL21	GTTGCCTCAAGTACAGCCAAA	GCTGCAAGAGAACTGAACAGACA
	AGAACAGGATAGCTGGGATGG	CGTGAACCACCCAGCTTGA
PDPN	GGAAGGTGTCAGCTCTGCTC	ACCGTGCCAGTGTTGTTCTG
	CGCCTTCCAAACCTGTAGTC	AGCACCTGTGGTTGTTATTTTGT
CD45	ACCACAAGTTTACTAACGCAAGT	AAACGATCGGTGACTTTTGG
	TTTGAGGGGGATTCCAGGTAAT	AGCTCTTCCCCTTTCCATGT
CD73	CCAGTACCAGGGCACTATCTG	GCAGCATTCCTGAAGATGCG
	TGGCTCGATCAGTCCTTCCA	CTCCCGAGTTCCTGGGTAGA
CD90	ATCGCTCTCCTGCTAACAGTC	TGCTCTCAGTCTTGCAGGTG
	CTCGTACTGGATGGGTGAACT	TGGATGGAGTTATCCTTGGTGTT
CD105	TGCACTTGGCCTACAATTCCA	CTGCCAATGCTGTGCGTGAA
	AGCTGCCCACTCAAGGATCT	GCTGGAGTCGTAGGCCAAGT
CXCL13	TATCCCTAGACGCTTCATTGATCG	CATAGATCGGATTCAAGTTACGCC
	CCATTCAGCTTGAGGGTCCACA	TCTTGGTCCAGATCACAACTTCA

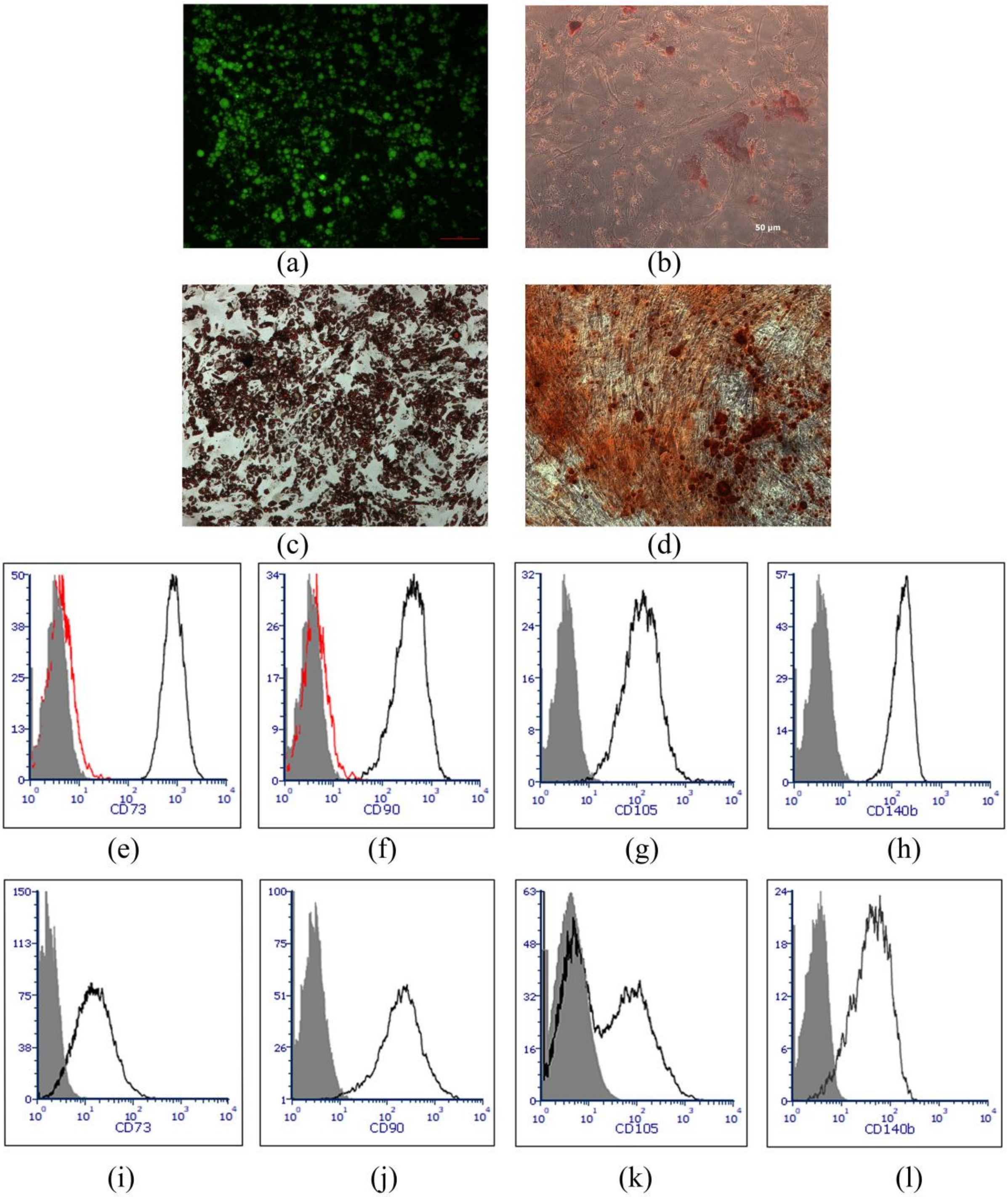

Figure S3: Validation of MSC Identity and Differentiation Potential. (a) adipogenic differentiation of mouse MSCs assessed on day 14 using BODIPY staining, highlighting lipid droplet accumulation (green fluorescence); (b) osteogenic differentiation of mouse MSCs confirmed by Alizarin Red staining, demonstrating calcium deposition in mineralized nodules; (c) adipogenic differentiation of human MSCs evaluated on day 14 using Oil Red O staining; (d) Osteogenic differentiation of human MSCs validated by Alizarin Red staining; (e-h) flow cytometry analysis of human MSC marker expression. (e) CD73 (black histogram) vs. hematopoietic marker CD45 (red histogram) and isotype control (gray histogram); (f) CD90 (black histogram) vs. endothelial marker CD31 (red histogram) and isotype control (gray histogram); (g) CD105 (black histogram) and isotype control (gray histogram); (h) CD140b (black histogram) and isotype control (gray histogram); (i-l) flow cytometry analysis of mouse MSC marker expression, including (i) CD73, (j) CD90, (k) CD105, and (l) CD140b, with black histograms showing marker expression and gray histograms representing isotype controls.
