# Supplementary figures and images for: Artificial Tertiary Lymphoid Structures: Exploring Mesenchymal Stromal Cells as a Platform for Immune Niche Formation
Source: Int J Mol Sci. 2024 Dec 11;25(24):13286. doi: 10.3390/ijms252413286 (PMC11676966; doi:10.3390/ijms252413286)

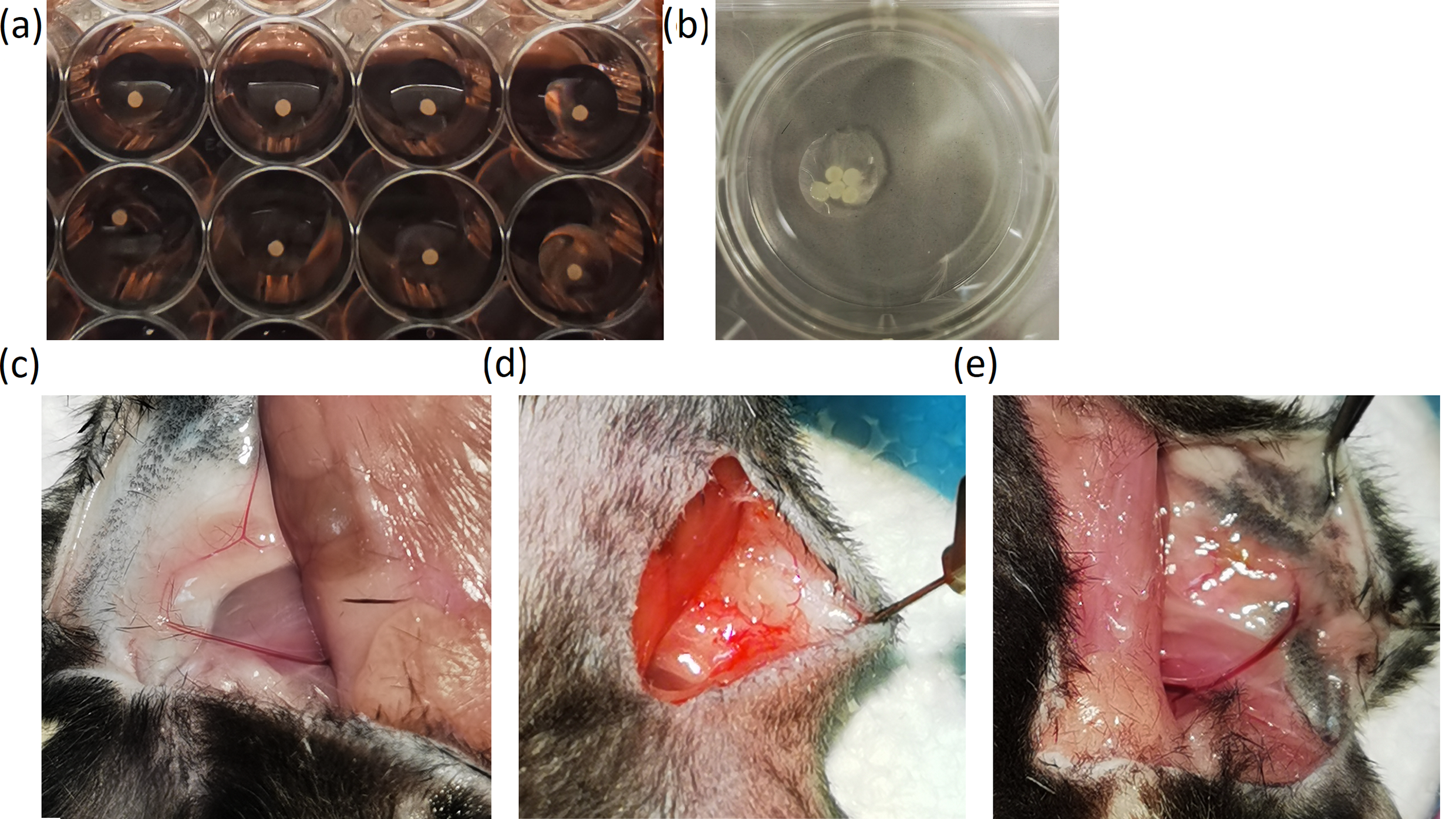

Supplement: Supplementary file 1 [file ijms-25-13286-s001.zip › S1.tif]

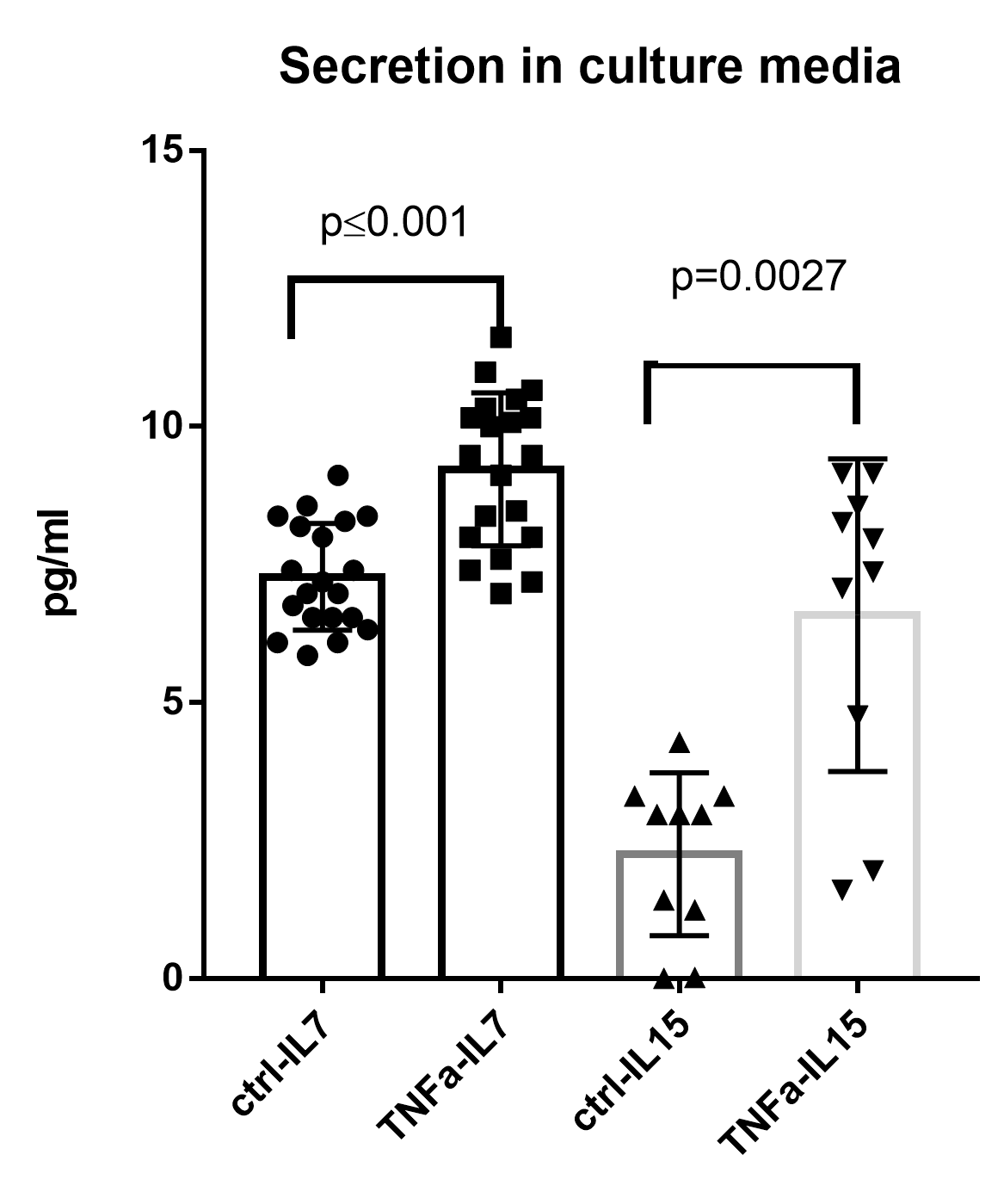

Supplement: Supplementary file 1 [file ijms-25-13286-s001.zip › S2.tif]
